# Supplementary material for: Dietary diversity insufficiently explains differences in prevalence of anaemia in pregnancy across regions in Nigeria: A secondary analysis of Demographic and Health Survey 2018
Source: PLOS Glob Public Health. 2025 May 29;5(5):e0004540. doi: 10.1371/journal.pgph.0004540 (PMC12121764; doi:10.1371/journal.pgph.0004540)
Supplement: S1 Table — (DOCX) [file pgph.0004540.s001.docx]

**Table S1: List of food groups used to determine minimum dietary diversity for the pregnant women**

| **Food group** | **Specific foods** |
| --- | --- |
| 1 | grains, white roots, tubers, plantain |
| 2 | pulses like beans, peas, lentils |
| 3 | nuts and seeds |
| 4 | dairy (milk and milk products) |
| 5 | meat, poultry, fish, small insects |
| 6 | Eggs |
| 7 | dark green leafy vegetables |
| 8 | vitamin A rich fruits/vegetables like mango, papaya, watermelon, tomato, carrots |
| 9 | other vegetables |
| 10 | other fruits |
