# Supplementary material for: Dietary diversity insufficiently explains differences in prevalence of anaemia in pregnancy across regions in Nigeria: A secondary analysis of Demographic and Health Survey 2018
Source: PLOS Glob Public Health. 2025 May 29;5(5):e0004540. doi: 10.1371/journal.pgph.0004540 (PMC12121764; doi:10.1371/journal.pgph.0004540)
Supplement: S2 Table — (DOCX) [file pgph.0004540.s002.docx]

**Table S2. Association between dietary diversity and anaemia among pregnant women nationally and across the six regions in Nigeria (n = 1,502)**

| **Region/minimum dietary diversity** | **Total** | **All women**  **(n = 1,502)**  **% (95%CI)** | **Anaemic**  **(n = 959)**  **% (95%CI)** | **Not anaemic**  **(n = 543)**  **% (95%CI)** | **p-value** |
| --- | --- | --- | --- | --- | --- |
| **Nigeria overall** |  |  |  |  |  |
| Not achieved (<5 food groups) | 807 | 54.2 (51.1 - 57.3) | 56.7 (53.0 - 60.2) | 50.4 (45.2 - 55.5) | **0.049** |
| Achieved (≥5 food groups) | 695 | 45.8 (42.7 - 48.9) | 43.3 (39.8 - 47.0) | 49.6 (44.5 - 54.8) |  |
| **North-Central** |  |  |  |  |  |
| Not achieved (<5 food groups) | 183 | 62.9 (57.0 - 68.4) | 62.8 (55.8 - 69.3) | 63.2 (52.8 - 72.5) | 0.949 |
| Achieved (≥5 food groups) | 104 | 37.1 (31.6 - 43.0) | 37.2 (30.8 - 44.2) | 36.8 (27.5 - 47.2) |  |
| **North-East** |  |  |  |  |  |
| Not achieved (<5 food groups) | 156 | 52.1 (45.6 - 58.5) | 53.3 (44.7 - 61.6) | 50.7 (40.8 - 60.6) | 0.703 |
| Achieved (≥5 food groups) | 145 | 47.9 (41.5 - 54.4) | 46.7 (38.4 - 55.3) | 49.3 (39.5 - 59.2) |  |
| **North-West** |  |  |  |  |  |
| Not achieved (<5 food groups) | 231 | 52.1 (46.5 - 57.6) | 54.9 (48.0 - 61.6) | 47.8 (39.3 - 56.4) | 0.193 |
| Achieved (≥5 food groups) | 221 | 47.9 (42.4 - 53.6) | 45.1 (38.4 - 52.0) | 52.2 (43.6 - 60.7) |  |
| **South-East** |  |  |  |  |  |
| Not achieved (<5 food groups) | 89 | 56.8 (48.1 - 65.0) | 53.9 (44.6 - 63.0) | 63.7 (48.6 - 76.4) | 0.299 |
| Achieved (≥5 food groups) | 89 | 43.3 (35.0 - 51.9) | 46.1 (37.0 - 55.4) | 36.3 (23.6 - 51.4) |  |
| **South-South** |  |  |  |  |  |
| Not achieved (<5 food groups) | 74 | 58.4 (48.9 - 67.4) | 63.5 (49.5 - 75.5) | 51.2 (38.5 - 63.7) | 0.191 |
| Achieved (≥5 food groups) | 56 | 41.6 (32.7 - 51.1) | 36.5 (24.5 - 50.5) | 48.8 (36.3 - 61.5) |  |
| **South-West** |  |  |  |  |  |
| Not achieved (<5 food groups) | 74 | 48.3 (37.2 - 59.5) | 56.2 (44.1 - 67.7) | 38.7 (22.7 - 57.6) | 0.109 |
| Achieved (≥5 food groups) | 80 | 51.7 (40.5 - 62.8) | 43.8 (32.4 - 55.9) | 61.3 (42.4 - 77.3) |  |

*Adequate refers to consumption of at least five food groups. Inadequate refers to consumption of less than five food groups. Chi square test compares anaemic and non-anaemic pregnant women.*

*Though, no association was found between dietary diversity and region, p >0.05 for all regions, an analysis of the country data revealed a significant association between these two variables, p = 0.049. Anaemia prevalence in pregnant women with inadequate dietary diversity was 56.7% (95%CI: 53.0-60.2) compared to those with adequate dietary diversity, 43.3% (95%CI: 39.8-47.0).*
