# Supplementary figures and images for: Dietary diversity insufficiently explains differences in prevalence of anaemia in pregnancy across regions in Nigeria: A secondary analysis of Demographic and Health Survey 2018
Source: PLOS Glob Public Health. 2025 May 29;5(5):e0004540. doi: 10.1371/journal.pgph.0004540 (PMC12121764; doi:10.1371/journal.pgph.0004540)

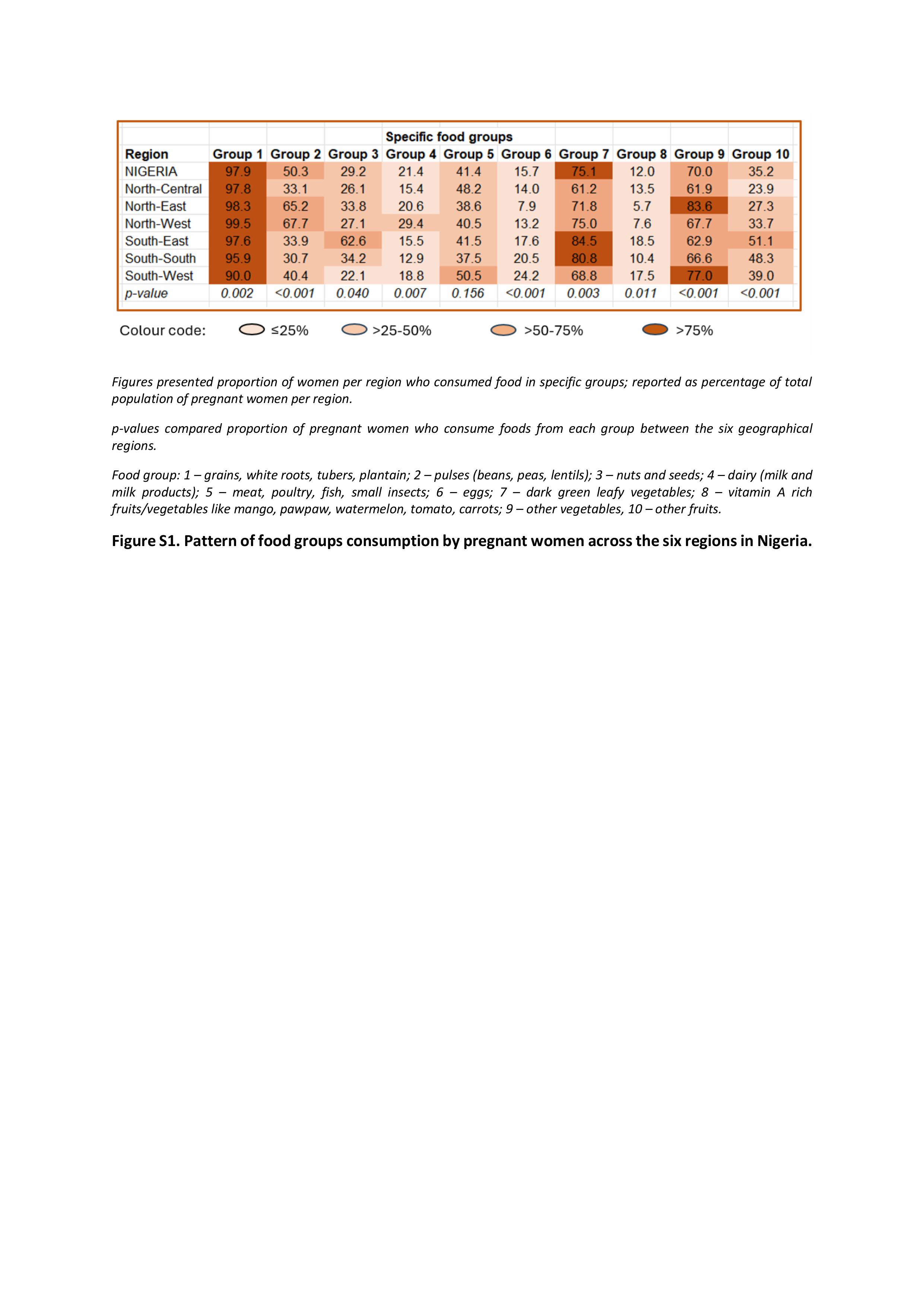

Supplement: S1 Fig — (TIF) [file pgph.0004540.s003.tif]
