# Supplementary material for: Dietary diversity insufficiently explains differences in prevalence of anaemia in pregnancy across regions in Nigeria: A secondary analysis of Demographic and Health Survey 2018
Source: PLOS Glob Public Health. 2025 May 29;5(5):e0004540. doi: 10.1371/journal.pgph.0004540 (PMC12121764; doi:10.1371/journal.pgph.0004540)
Supplement: Attachment — Submitted filename: RESPONSE TO REVIEWERS.pdf [file pgph.0004540.s005.pdf]

**PGPH-D-24-02093**
**Dietary diversity insufficiently explains differences in prevalence of anaemia in pregnancy across regions in Nigeria: a secondary analysis of Demographic and Health Survey 2018**
**PLOS Global Public Health**

Dear Editor,
We thank you and the reviewers for the valuable time spent in the critical review of this manuscript which no doubt has improved its quality. The comments were all noted and have been addressed with point-by-point response to each comment below (in blue font).
***Please note that all page and line numbers listed in our responses are of the revised manuscript with track changes (some highlighted yellow).***

Thank you.
Best regards,
Ochuwa A. Babah
Corresponding Author

Journal Requirements:

1. Please provide separate figure files in .tif or .eps format.
For more information about figure files please see our guidelines:
https://journals.plos.org/globalpublichealth/s/figures
https://journals.plos.org/globalpublichealth/s/figures#loc-file-requirements
**Response**
This is noted. All figures are now in tif format.

2. We do not publish any copyright or trademark symbols that usually accompany proprietary names, eg (R), (C), or TM (e.g. next to drug or reagent names). Please remove all instances of trademark/copyright symbols throughout the text, including ® on Figure 1.
**Response**
Thank you for this comment. This is noted. Copyright symbol has been removed from figure 2 which is the map of Nigeria. Figure 1 is the flow diagram and did not have a copyright symbol.

3. Please provide an Author Summary. This should appear in your manuscript between the Abstract (if applicable) and the Introduction, and should be 150–200 words long. The aim should be to make your findings accessible to a wide audience that includes both scientists and non-scientists. Sample summaries can be found on our website under Submission Guidelines:
https://journals.plos.org/globalpublichealth/s/submission-guidelines#loc-parts-of-a-submission.
**Response**
We have now included a plain language summary between the abstract and the introduction as shown below. This can be found in the manuscript on Page 3, lines 50-64.

**Plain language summary**

Anaemia in pregnancy is a reduction in the red blood cells that carry oxygen within the body during pregnancy. It is a global health issue and is known to be among the top ten leading causes of maternal death. About six out of ten pregnant women in Nigeria have anaemia, but this varies from region to region. If not promptly diagnosed and treated, anaemia may harm the health of the mother and/or her baby. Previous studies have identified diet as a risk factor for anaemia in pregnancy. We are aware that women's diet is influenced by factors like type of foods available, cultural practices including food taboos, and the household's purchasing power, which in turn influences the quantity and quality of food consumed. These factors affect the dietary diversity and can affect whether pregnant women develop anaemia. This study investigated whether the diversity of food groups women consume could explain why the occurrence of anaemia differs across regions in Nigeria. We found that diet alone does not explain the regional differences in anaemia occurrence, suggesting that there are other factors which may better explain the difference in anaemia's occurrence across regions of Nigeria.

4. Figure 1: please (a) provide a direct link to the base layer of the map (i.e., the country or region border shape) and ensure this is also included in the figure legend; and (b) provide a link to the terms of use / license information for the base layer image or shapefile. We cannot publish proprietary or copyrighted maps (e.g. Google Maps, Mapquest) and the terms of use for your map base layer must be compatible with our CC-BY 4.0 license.

Note: if you created the map in a software program like R or ArcGIS, please locate and indicate the source of the basemap shapefile onto which data has been plotted.

If your map was obtained from a copyrighted source please amend the figure so that the base map used is from an openly available source. Alternatively, please provide explicit written permission from the copyright holder granting you the right to publish the material under our CC-BY 4.0 license.

Please note that the following CC BY licenses are compatible with PLOS license: CC BY 4.0, CC BY 2.0 and CC BY 3.0, meanwhile such licenses as CC BY-ND 3.0 and others are not compatible due to additional restrictions.

If you are unsure whether you can use a map or not, please do reach out and we will be able to help you. The following websites are good examples of where you can source open access or public domain maps:
* U.S. Geological Survey (USGS) - All maps are in the public domain. (http://www.usgs.gov)
* PlaniGlobe - All maps are published under a Creative Commons license so please cite "PlaniGlobe, http://www.planiglobe.com, CC BY 2.0" in the image credit after the caption. (http://www.planiglobe.com/?lang=enl)
* Natural Earth - All maps are public domain. (http://www.naturalearthdata.com/about/terms-of-use/)

**Response**

This is noted. We have replaced the first map with another created on ArcGIS Pro V 3.3 and cited the source with the URL. Figure 2. This new map has a CC BY 4.0 licence. We acknowledged a colleague who assisted in creating the map of Nigeria as follows:

"Appreciations to Peter M. Macharia of Institute of Tropical Medicine, Antwerp, Belgium who helped create the map of Nigeria on ArcGIS Pro V 3.3". Page 25, lines 450-452.
Source: This map was plotted by the author in ArcGIS Pro V 3.3 (ESRI, Redlands, CA, USA) based on boundaries from GRID3 under a CC BY 4.0 License at:
https://data.grid3.org/datasets/GRID3::grid3-nga-operational-lga-boundaries/about

**Additional Editor Comments**
The authors need to revised the following points:

Methods: Details of ethics committee are missing.

**Response**

Thank you for drawing our attention to this omission of the name of the ethics committees from which approval was obtained for the DHS. Details on ethics committee has now been included thus "The DHS received ethical approval for the survey protocol from the National Health Research Ethics Committee of Nigeria (NHREC) and the ICF Institutional Review Board before conducting the survey" on Page 11, lines 240-241.

Results:
Table 1: The authors have define anemia Anemic (Hb>=11 g/dl) which is not correct. Not sure if the data is appropriate for anemic or nonanemic women. The proportions should indicate prevalence of anemia in the given subgroups. Once anemic prevalence is presented, there is no need for non-anemia prevalence. This is overlapping data.

**Response**

Thank you for flagging this important typographical error on Table 1. Anaemic is <11g/dl and non-anaemic is ≥11g/dl. We have made necessary corrections on Table 1, Page 12. We also cross-checked the do-file and did not find any error in the Stata coding in this regard. In addition, we have deleted data for non-anaemic group, leaving the data for anaemic pregnant women. Table 1 on Page 13 and 14.

Table 2: The data should be shown trimester wise for better granularity.

**Response**

Thank you for your suggestion. We have included data by trimester on Table 2. Page 16. We also added the following to the result text "Looking at effect of trimester on anaemia and haemoglobin concentration, *Table 2* shows that the prevalence of anaemia increases significantly as trimester advances, with lowest prevalence of 44.7 (95%CI: 39.1 - 50.4) in the first trimester and highest prevalence of 69.4 (95%CI: 64.5 - 74.0) in the third trimester (p <0.001). The mean haemoglobin concentration drops significantly as trimester advances with highest values in the first trimester and lowest values in the third trimester in both general population of pregnant women and in sub-group of pregnant women with anaemia (p <0.001 and p = 0.02 respectively)." Page 15, lines 270-277.

Figure 3: The proportions of mild, moderate and severe anemics do not add to the total anemia prevalence. E.g. North central - anemia in 69.4%. However, mild and moderate

anemic fractions add to 97.3%.

**Response**

Thank you for your valuable comment. We have expanded the footnote by adding the following: “Prevalence of all anaemia is based on total number of pregnant women in the sample both anaemic and non-anaemic as the denominator whereas prevalence presented for anaemia severity (mild, moderate and severe) is based on total number of women that are anaemic as denominator”, Figure 3 footnote. The prevalence of anaemia severity adds up to 100%, e.g. for North Central, 48.9% have mild, 48.3% moderate and 2.8% severe as the denominator is total number of anaemic pregnant women. This is independent of the 69.4% which is the anaemia prevalence, which denominator is total number of pregnant women and comprised both anaemic and non-anaemic. We have modified the figure by presenting all anaemia prevalence before the subgroups of anaemia severity in the bar charts.

Table 3: For logistic regression, the reference should not be extreme in the range. Rather a midpoint should be used. E,g, for wealth quantile, it is better to use middle as reference than poorest.

**Response**

Thank you for your suggestion. We prioritized an extreme category, poorest, as our reference category for wealth quintile based on our research question and because this allows for an easier, more straightforward and more meaningful interpretation of the odds ratio and makes it easier to understand the trend in the association, compared to using a middle category.

Discussion: The authors needs to add details of the food groups which were not adequately represented that might be associated with increased risk of anemia.

**Response**

Your comments are well noted. A comment has been made in the Discussion identifying food groups not commonly consumed in Nigeria as found in this study, that might have an impact on dietary diversity and thus the prevalence of anaemia in pregnancy. We added the paragraph “In this study, we observed that most of the pregnant women did not consume foods in group 6 (eggs) and 8 (vitamin A-rich fruits and vegetables like mango, pawpaw, watermelon, tomatoes and carrots). This might be due to seasonal unavailability of the foods, and can have an impact on dietary diversity and thus the prevalence of anaemia in pregnancy. Previous studies found that consumption of eggs, which are known to contain some amounts of heme and non-heme iron, improve haemoglobin concentration and serum ferritin levels.[32, 33] Vitamin A deficiency has also been associated with anaemia in pregnancy.[34, 35] Vitamin A plays a role in erythropoiesis by facilitating iron mobilization. In a state of deficiency, it causes an impairment in iron mobilisation from the liver and spleen, making it unavailable for erythropoiesis resulting in anaemia.[36]” Page 21, lines 355-366. We also included the corresponding references, 32-36 cited in-text.

References


32. Azmandian J, Shamspour N, Azmandian A, Ahmadipour H, Alinaghi Langari T. The Effect of Egg White Meal on Anemia in Patients on Hemodialysis Taking Erythropoietin and Iron Infusion. Journal of Renal Nutrition. 2024.

33. Rofiah K, Nirwana BS, Damayanti I, Mawarti KS. The Effect of the Grant of a Boiled Egg to Increased Levels of Hemoglobin (Hb) in Pregnant Women with Anemic at Cerme Village Health Center Kediri Regency in 2022. Journal for Quality in Public Health. 2022;6(1):24-30.
34. Radhika MS, Bhaskaram P, Balakrishna N, Ramalakshmi BA, Devi S, Kumar BS. Effects of vitamin A deficiency during pregnancy on maternal and child health. Bjog. 2002;109(6):689-93.
35. Hamdy AM, Abdel Aleem MM, El-Shazly AA. Maternal Vitamin A Deficiency during Pregnancy and Its Relation with Maternal and Neonatal Hemoglobin Concentrations among Poor Egyptian Families. International Scholarly Research Notices. 2013;2013(1):652148.
36. Mejia LA, Erdman Jr JW. Impact of Vitamin A Deficiency on Iron Metabolism and Anemia: A Historical Perspective and Research Advances. Nutrition Reviews. 2024.

Conclusion: The conclusion is presently very generic. The author need to specify the exact factors related to dietary diversity and demographic characteristics that are significantly associated with increased risk of anemia.

**Response**

Thank you for this valuable comment. To address the comment on factors related to dietary diversity, we have briefly mentioned demographic variables that are associated with increased risk of anaemia thus: "Although, sociodemographic and obstetric factors like being single, being in second or third trimester of pregnancy and having lower level of education were associated with increased risk of anaemia in pregnancy, our findings suggest that there are likely still are other factors such as food security in terms of availability and accessibility and the seasonality of food availability which we could not account for in this study, including ...........", Page 24, lines 433-437 and Page 25, lines 438-439.

**Reviewers' comments**

Reviewer #1: Introduction

1. Please talk in brief about dietary diversity in Nigeria and how it is defined

**Response**

Your comments are well noted. The definition of dietary diversity globally has been added to the introduction as follows: "Dietary diversity is defined as "the number of different food groups consumed over a given reference period".[15] Page 5, lines 96-97. This definition was also referenced and the new reference 15 has been added to the reference list. We do not have a separate local definition for dietary diversity in Nigeria.

We also added the sentence "Evidence suggests that dietary diversity in Nigeria is poor, with only half of reproductive aged women and less than one-quarter of children have adequate dietary diversity".[22] Page 5, lines 103-105.

References

15. Ruel MT. Operationalizing Dietary Diversity: A Review of Measurement Issues and Research Priorities. The Journal of Nutrition. 2003;133(11):3911S-26S.
22. Akseer N, Tasic H, Adeyemi O, Heidkamp R. Concordance and determinants of mothers' and children's diets in Nigeria: an in-depth study of the 2018 Demographic and Health Survey. BMJ Open. 2023;13(7):e070876.

Methods:

1. Use consistent terminology: reproductive-age women OR women aged 15-49.

**Response**

Your comment is well noted. Under study population we have changed "women aged 15-49" to "reproductive age women" to ensure consistency. Page 7, line 147. We have also defined reproductive age at first mention by putting "15-49 years" in bracket. Page 5, line 104.

2. 15-49 unit is missing?

**Response**

Your comment is noted. We have added the unit "years" where this occurs, now at first mention of reproductive age. Page 5, line 104.

3. Line 110 to 114 please move to study population section.

**Response**

Thank you for the advice. The sentence "The survey used a multistage sampling technique to select a total 42,000 households; in one-third of the sampled households (14,000), reproductive-age women were screened for anaemia, and those pregnant women among this sub-sample were included in this study." has been moved to the study population section. Page 7, lines 149-153.

4. Line 118 says providing consent… it should be providing written informed consent

**Response**

Thank you. This has been corrected. Now on Page 7, line 148.

5. Section on study area and its characteristics is missing

**Response**

Your comment is noted. A section on study location has been included in the methods section. Page 7, lines 138-144. We have written in this section: "Nigeria is a sub-Saharan African country with a population of over 227 million as of 2023.[25] It is bounded by the Republic of Niger in the North, by Cameroon and Chad Republic on the East, by Benin Republic on the West and by the Gulf of Guinea in the Atlantic Ocean in the South.[26] The country is divided into six geopolitical zones or regions.[26] It is a country with diverse ethnicity, with about 250 ethnic groups.[26] Its economy is one of the largest in sub-Saharan Africa.[26] We have also added two new references.

References:

25. World Bank Group. Population, total - Nigeria. 2025. Accessed: 8 February 2025. Available from: https://data.worldbank.org/indicator/SP.POP.TOTL?locations=NG.
26. Udo, Reuben Kenrick, Kirk-Greene, Anthony Hamilton Millard, Ajayi JFA, Falola, et al. Nigeria. Encyclopedia Britannica. 2025. Accessed 8 February 2025. Available from: https://www.britannica.com/place/Nigeria.

6. Who and how data was collected in DHS is not mentioned. Hard copy or e-data collection? What about data quality check? How data managed?

**Response**

Your comments are well noted. A section on data collection has been created and reads: "Data were collected on electronic devices by trained enumerators. For biomarkers which included haemoglobin concentration measurements, the data was first recorded on paper questionnaires and subsequently entered into the electronic database. Quality assurance strategies put in place before and during the survey included training and re-training of all the research team members. The enumerators were trained on how to enter data on the electronic platform, how to use the biomarker checklist and how to collect specimens for investigations. Anthropometric measurements suspected to be incorrect were flagged and re-measurement performed. Six quality control officers were nominated, and together with the state coordinators, they monitored the fieldwork to ensure compliance with the protocol and research ethics and to promptly identify issues as they occurred." Page 8, lines 161-172.

7. Pregnant women height and weight data would have added value. If data is available, please include

**Response**

Thank you. We have data for weight and height. We have included these on Table 1, Page 14.

8. Morbidity data?

**Response**

Unfortunately, we do not have data for this. Thank you.

9. Which ethical committee approval were sought?

**Response**

Ethical approval was obtained from the National Health Research Ethics Committee of Nigeria (NHREC) and the ICF Institutional Review Board. This has now been included in the manuscript on Page 11, lines 240-241.

10. Cite reference for wealth quintile (poorest, poorer, middle, richer and richest)

**Response**

The reference 29 has now been included. Page 9, line 207.

Reference

29. DHS. Wealth index. The DHS Program. 2016. Accessed: 6 February 2025. Available from: https://dhsprogram.com/topics/wealth-index/.

Results:

1. How many women available in the said population? How many of it approached? Consented? Screened? Enrolled? Screen failure? Withdrawal? Please mention this in very first para of results section.

**Response**

Thank you for bringing this observation to the fore. This information has been included in the first paragraph of the result section on Page 11, lines 248-251 as follows: "A total of 15,236 reproductive aged women invited for anaemia screening. Of the women, 486 did not have anaemia screening done because they either refused screening (n=359), did not report for screening (n=124) or gave other reasons (n=3). A total of 14,750 were screened for anaemia, of which only 1,525 were pregnant women, *Figure 1*".

2. Table 1, age groups- 15-20 and 20-29 are overlapping, please rectify

**Response**

Thank you for drawing attention to this typographical error which has now being correction on Table 1. Page 13. We also cross-checked in the do-file and confirmed that <20 years was coded as the youngest age group.

3. How women below 18 years ages were consented for the study?

**Response**

Thank you. According to the NDHS report, "Women age 15-49 were also eligible for haemoglobin testing. Children aged 6-59 months were also eligible for haemoglobin testing. Blood specimens for the tests were collected from eligible women who voluntarily consented to be tested and from all children age 6-59 months for whom consent was obtained from their parents or the adult responsible for them". . The DHS obtained consent directly from the women and this is likely because they were considered to be emancipated as per the national guideline drawn up by the Federal Ministry of Health, Nigeria, to ensure inclusivity. It was included in the section on ethics that the women gave informed consent, Page 11, lines 243-244.

Reference


- FMOH. A Consensus Report on Guidelines for Young Persons' Participation in Research and Accessing Sexual and Reproductive Health Services in Nigeria. Federal Ministry of Health, Nigeria. 2014. Available from: https://www.popcouncil.org/uploads/pdfs/2014HIV_YoungPersonsSRH-Nigeria.pdf.


4. Table 2, column heading % is mentioned, however CI missing

**Response**

Thank you for highlighting this omission. 95%CI has now been added to the affected column heading. Table 2 on Page 16.

5. Figure S1 and 4 there is no consistency in which food groups reported/mentioned. Please rectify

**Response**

Thank you for this comment. Figure S1 and Figure 4 are addressing two different themes. Figure S1 is talking about specific food groups, while Figure 4 is talking about the number of food groups consumed previous day (dietary diversity score). We have added “dietary diversity score” in bracket to Figure 4 column heading to make the difference more striking to potential readers. And we have added the word specific to the column heading for figure S1 which now reads specific food group and the numbers 1, 2, 3, etc now reads group 1, group 2, group 3, etc.

6. Figure 4, why data for only 1502 presented?

**Response**

Thank you. This lower sample size 1502 is due to missingness in the variable dietary diversity, as there were 23 women with missing data in this variable. We have included in the Analysis section that “Complete case analysis was done; no imputation technique was applied to handle missing data”, Page 11, line 235-236.

7. Figure 4 showed data for overall Nigeria as well, but figure S1 showed data for regions only

**Response**

Thank you for bringing this observation forth. We have now included data for overall Nigeria in Figure S1 for consistency. Both Figures 4 and S1 now have data for overall Nigeria and for specific regions. We have now included a column in Figure 4 showing mean dietary diversity score across regions with corresponding p-value comparing the score across the six regions.

8. Figure S1, p value given for each food group, however, in figure 4, p value overall given?

**Response**

Thank you for bringing this observation forth. Considering that figure S1 looked at specific food groups which were analysed as binary variables (yes/no), it was possible to test for association for individual food groups versus region. On the contrary, we could not determine association for each score for Figure 4 by region because each MDD score is not binary, that is the variable is an only one column category and thus statistics could not be computer to determine association. It was only possible to run analysis with MDD score (which has 11 levels from 0 to 10) as a single variable and region (which has six levels) as the second variable.

**NEW ADDITION TO MANUSCRIPT**

1. We added “Centre for Clinical Trials, Research and Implementation Science, College of Medicine, University of Lagos, Idi-Araba, Lagos, Nigeria” as a second affiliation for Bosede Bukola Afolabi. Page 1, lines 12-13.
2. Under Acknowledgment, we have acknowledged Peter M. Macharia for assisting in creating the map of Nigeria on ArcGIS Pro V 3.3”. Page 25, lines 450-452.
