# Supplementary material for: Dietary diversity insufficiently explains differences in prevalence of anaemia in pregnancy across regions in Nigeria: A secondary analysis of Demographic and Health Survey 2018
Source: PLOS Glob Public Health. 2025 May 29;5(5):e0004540. doi: 10.1371/journal.pgph.0004540 (PMC12121764; doi:10.1371/journal.pgph.0004540)
Supplement: Attachment — Submitted filename: Response_to_reviewers_auresp_2.pdf [file pgph.0004540.s006.pdf]

PGPH-D-24-02093

Dietary diversity insufficiently explains differences in prevalence of anaemia in pregnancy across regions in Nigeria: a secondary analysis of Demographic and Health Survey 2018

PLOS Global Public Health

Dear Aditi Apte,
Academic Editor,
PLOS Global Public Health.

Thank you for the valuable time you and the reviewers have spent to critically appraise this manuscript. We have addressed the review comments and provided a summary as a point-by-point response below. Page numbers cited are of the manuscript with tracked changes.

Thank you.
Best regards,
Ochuwa A. Babah
Corresponding Author

Review comments

1. Table 1 and table 2 : Please specify the relevance of the p values in the footnote. Are these p values for multiple group comparison.

Response

Thank you for bringing this to our notice. We have specified as footnotes what the p-values in Tables 1 and 2 compare (Page 13, lines 253-254 and Page 15, line 273 respectively).

2. Table 3: For wealth quantiles, poorest is used as a reference for the logistic regression. Poorest is an extreme category. Please use middle as the reference.

Response

The logistic regression analysis has been re-run using middle category as the reference for wealth index. Necessary adjustment has been made to the crude and adjusted values accordingly, and other variables confirmed in the multivariable model to be the same. The goodness of fit, tolerance and variance inflation factor reported in the footnote remained unchanged. (Pages 18-19). Thank you.
